# Impairment of intellectual functions after surgery and posterior fossa irradiation in children with ependymoma is related to age and neurologic complications

**DOI:** 10.1186/1471-2407-8-15

**Published:** 2008-01-21

**Authors:** Katja von Hoff, Virginie Kieffer, Jean-Louis Habrand, Chantal Kalifa, Georges Dellatolas, Jacques Grill

**Affiliations:** 1 Department of paediatric and adolescent oncology, Gustave Roussy institute, 39 rue Camille Desmoulins, 94805 Villejuif cedex, France; 2 Childrens university hospital Wuerzburg, Josef-Schneider str.2, 97080 Wuerzburg, Germany; 3 Ressource center for patients with brain injuries, national rehabilitation hospital, 14 rue du val d'Osne, 94415 Saint-Maurice, France; 4 Department of radiotherapy, Gustave Roussy institute, 39 rue Camille Desmoulins, 94805 Villejuif cedex, France; 5 Laboratory of psychology and cognitive neurosciences, CNRS-FRE 2987, 71 avenue Edouard Vaillant, 92774 Boulogne-Billancourt, France

## Abstract

**Background:**

To investigate the neuropsychological outcome of children treated with surgery and posterior fossa irradiation for localized infratentorial ependymoma.

**Methods:**

23 patients (age 0.3 – 14 years at diagnosis) who were treated with local posterior fossa irradiation (54 Gy) underwent one (4 patients) or sequential (19 patients) neuropsychologic evaluation. The last evaluation was performed at a median of 4.5 (1 to 15.5) years after RT.

**Results:**

Mean last full scale IQ (FSIQ), verbal IQ (VIQ) and PIQ were 89.1, 94.0, and 86.2 respectively. All patients had difficulties with reading, and individual patients showed deficits in visuospatial, memory and attentional tasks. There was no trend for deterioration of intellectual outcome over time. All 5 children with IQ scores ≤ 75 were under the age of four at diagnosis. There was a significant association between the presence of cerebellar deficits and impaired IQ (72.0 vs 95.2, p < 0,001). The absence of hydrocephalus was an indicator of better neuropsychologic outcome (mean FSIQ of 102.6 vs 83.9, p = 0.025).

**Conclusion:**

Within the evaluated cohort, intellectual functions were moderately impaired. Markedly reduced IQ scores were only seen with early disease manifestation and treatment, and postoperative neurological deficits had a strong impact on intellectual outcome.

## Background

Within the posterior fossa, ependymoma is the second most common malignant tumour in children [1]. As with other paediatric central nervous system (CNS) tumours, finding the balance between effective treatment and preservation of psychomotor development is challenging. Modern approaches aim at maximizing surgical resection while reducing the volume of irradiation since complete tumour removal is the main prognostic factor [2-4].

As a consequences of brain damage caused by the tumor itself and the surgery, some children develop neurologic deficits such as cerebellar dysfunction and cranial nerve palsies [5]. Indeed, radiation therapy rarely causes neurologic damage in the absence of complications such as radionecrosis or stroke. More aggressive surgery may thus increase the risk of neurologic damage.

Progressive intellectual impairment is a serious side effect of whole brain irradiation [6-9], the extent to which intellectual capacities are also diminished due to local radiation to the posterior fossa remains to be determined. Intellectual quotient (IQ) is preserved in patients with ependymoma after posterior fossa irradiation only, compared to children with medulloblastoma who received craniospinal irradiation (CSI) [10]. Furthermore preliminary data suggest that there may be only limited decline in neurocognitive functions after local posterior fossa irradiation [3,11].

To determine the risk factors for intellectual impairment and to define the neuropsychological profile of long term survivors of localised infratentorial ependymoma we analysed the long-term neuropsychological outcome of children who received posterior fossa radiotherapy in a cohort of patients treated between 1986 and 2003 either at diagnosis (in children over 5 years of age) or after first relapse following chemotherapy in younger children. Patients who were diagnosed 1998 and later were evaluated prospectively.

All potential risk factors for intellectual impairment [12] were studied, including pre-operative complications such as hydrocephalus, surgical complications and persistent cerebellar deficits, age, and radiation volume (conformational versus whole posterior fossa)

## Methods

### Patients

Patients were included in this study if they (i) were diagnosed and operated on a localised infratentorial ependymoma, (ii) received local posterior fossa irradiation at the Institute Gustave-Roussy in Villejuif between 1986 and 2003, as initial treatment or after chemotherapy according to the BBSFOP protocol (Carboplatin/Procarbazin; Etoposide/Cisplatin; Vincristine/Cyclophosphamide) [13], (iii) had at least one standardised neuropsychologic evaluation, and (iii) had no abnormal premorbid psychomotor development as reported by the parents.

Twenty-three patients fulfilled these criteria. Informed consent was obtained from all patients. Patient characteristics are shown in table 1. Age at diagnosis ranged between 0.3 and 14.2 years (median 7.2). Of ten patients who were under the age of five at diagnosis, eight were irradiated under the age of five, three of them were irradiated before the age of three. There was a male predominance with 17 boys within the group. 16 patients were presenting with signs of intracranial pressure at disease manifestation. All patients had surgical resection with gross total resection achieved in 18 patients. Four patients received postoperative chemotherapy according to the French BBSFOP protocol [13] and commenced to radiotherapy due to progression of residual tumour or relapse.

**Table 1:** General characteristics of 23 patients included in the study.

Age at diagnosis	0.3 – 14.2 y (median 7.2)
Male gender	17
Pts under 5 y at diagnosis	10
Pts under 5 y at irradiation	8
Preradiation chemotherapy	4
Hydrocephalus at presentation	16
Gross total resection at 1^st ^surgery	18
Second surgery	4
Radiation therapy dose	50–62 Gy
Opposite lateral beams	12
Conformal irradiation	11
Postoperative cerebellar mutism	0
Postoperative cerebellar syndrome	15
Severe	3
Moderate	7
Mild	5
Cerebellar syndrome at last IQ evaluation	6
Severe	2
Moderate	2
Mild	2
Interval between RT and last IQ evaluation	1–15.5 y (median 4.5)
Age at last IQ evaluation	4.5–19.6 y (median 13.2)

RT = Radiotherapy.

IQ = Intellectual Quotient.

Pts = Patients.

Gy = Gray.

y = years.

### Radiotherapy

All patients were treated using megavoltage equipments (4.5 to 20 MV photons of a linear accelerator). Total dose ranged between 50 and 62 Gy, administered in 5 weekly sessions of 1.8 Gy per day, with each beam treated every day. The highest doses correspond to patients with gross residual disease present at the time of radiations. A computerized dose-distribution was made available in all patients using the DOSIGRAY^® ^software. In early patients, it was based on radiographic simulation films with hand-drawn tailored shieldings, based on physician knowledge of the anatomical landmarks, and tumour characteristics. More recently, a 3D high definition CT-scan based representation of dose-distribution superimposed with the posterior fossa structures, and tumour contour was made available. Dose-volume histograms for structures of interest were also generated. As far as technical considerations, early patients were treated in a straightforward approach combining two opposed laterals; recent 3D simulation, allowed conformation to the target with optimal sparing of adjacent organs (mainly pituitary, cochleas, chiasm). The gross tumour volume (GTV) for the primary site boost included the postoperative tumor bed. The clinical target volume (CTV) included the GTV, with an anatomically confined margin of 2 cm in the adjacent brain, whereas the planned target volume (PTV) expanded the CTV with a geometric margin of 1 cm. Multiple beams arrangements were used, ie 2 to 4 wedge anterior and/or posterior obliques. The early approach induced full dose of radiations in the entire posterior fossa, along with occipital and posterior temporal lobes. Only pituitary located at anterior margin, was kept to an acceptable level. The recent approach allowed marked reduced maximal dose to most structures outside the posterior fossa, including cochleas occipital and parietal lobes. The reverse side is that doses to the pituitary as well as integral dose to the temporal lobes were slightly increased due to beams exits.

### Neuropsychologic evaluation

A battery of age adapted standard neuropsychological tests was applied to all patients. This included an IQ measure using Wechsler scales WAIS-R for adults, WISC-III for children ≥ 7 years and WPPSI-R for children aged < 7 years [14,15]. WISC-III consists of 10 obligatory and 3 optional subtests with a range of test scores between 1 and 19 (average: 10). Complementary tests were used to describe patients neurocognitive abilities as previously described by our group [16]. Additionally reading skills were measured by using the test of the alouette [17]. Executive functions were evaluated using the Wisconsin card sorting test (WCST). The evaluation was completed by the judgment of line orientation [18], facial recognition [19], a copy of the Rey – Osterrieth complex figure for children over 7 and analysis of fine motor skills with the Purdue pegboard test [20]. This latter test evaluates fine motor speed with the dominant and non-dominant hand both separately and together. The tests were timed, and a period of three hours was allowed for the entire evaluation. They were always performed in the same order. Information regarding school placement, both before disease onset and at the time of the neuropsychological evaluation, was also collected from parent's interview.

Tests were performed longitudinally in 19 patients. Of them 13 patients were evaluated prospectively and had baseline evaluation within the first year after the completion of radiotherapy. One of them was too young for WPPSI-R and received K-ABC [21]. Six patients were first tested >1 year after completion of radiotherapy (1.1–11.6 years, median 7.6). Four patients had only one neuropsychological evaluation between 3.9 and 8.6 years after completion of RT (median 7).

Presence of cerebellar syndrome (Ataxia, Dysmetria, Nystagmus) was graduated as mild, moderate, or severe according to the impact on daily activities by an independent physician unaware of the neuropsychological performance using Riva's rating scale [22].

All patients were regularly screened for endocrinologic deficits and hearing impairment.

### Statistical Analysis

Statistical analysis was conducted using SPSS software (12.0 Version). Test results of the neuropsychological test (except IQ measures and subgroups) were normalised and transferred into Z-scores where score >= 2 corresponds to a probability of 95% to be outside of normal distribution.

The neuropsychologic profile was analysed descriptively based on the results of Wechsler subtests and the above mentioned additional test.

For analysis of risk factors for intellectual impairment, patients were divided into groups according to: age at radiotherapy (<5 y vs. ≥ 5 y); cerebellar syndrome, fine motor achievment; hydrocephalus at presentation, radiotherapy volume (conformal vs. posterior fossa). For each patient the result of the last FSIQ test was used. Comparison was done using Mann-Whitney-U test for non-parametrical data.

The age limit of 5 years was chosen due to reasons of clinical practice. Patients below 5 years of age were eligible for adjuvant BBSFOP chemotherapy [13]. Patients aged 5 or older would receive immediately adjuvant radiotherapy according to our institutional standard. Influence of age at RT was also analysed using linear regression.

Due to the small group size a multivariable analysis of risk factors was not reasonable and was therefore omitted.

Longitudinal data of achievment (FSIQ and reading) were analysed descriptively. Due to the small sample size and limited reliability of potential findings a random coefficient model was not used.

## Results

The last neuropsychologic evaluation was done at a median of 4.5 years after the completion of radiotherapy (range 1 – 15.5 years). At the last testing mean full scale intelligence quotient (FSIQ), verbal IQ (VIQ) and performance IQ (PIQ) were 89.1 (standard deviation SD 14.6), 94.0 (SD 12.4), and 86.2 (SD 16.1). Of the 23 evaluable patients FSIQ was 90 or above in 10 patients (43%), between 80 and 90 in eight patients (35%), and below 80 in five patients (22%).

### Profile of neuropsychological evaluation

In most of the WISC III subtests, scores were within average limits with mean scores above or equal 8 in 10 of 13 subtests (norms are 10 +/- 2 for each subtest). None of the patients showed a significant (≤ 2 SD) impairment of VIQ. Of the VIQ subtests 2 children achieved low test scores in the "information" subtest testing general knowledge. Four patients showed significant impairments in the optional memory subtest. PIQ was below VIQ in 17 patients with a mean difference of 7.8 points and a significant impairment in 6 patients. A marked impairment was seen in the chessboard/coding subtest and limited impairments in object assembly, symbols and picture arrangement, subtests evaluating speed of written performance and the capacity of visuo-spatial observation and organisation, respectively. Mean processing speed was also reduced. Table 2 shows the IQ subtest scores.

**Table 2:** Wechsler scale (WISC-III) subtest results of 23 patients.

	Number of patients with scores below minus 2 SD	mean
Full scale IQ	2/23	89.1
Verbal IQ	0/23	94.0
Performance IQ	6/23	86.2
Verbal comprehension	2/22	94.5
Perceptive organization	1/22	92.1
Speed	4/22	86.6
Verbal subtests		
Information	2/23	8.4
Similarities	0/23	9.3
Arithmetics	0/23	9.8
Vocabulary	0/23	9.1
Comprehension	1/23	9.4
Memory	4/22	8.0
Performance subtests		
Picture completion	3/23	9.4
Codes	6/22	5.9
Picture arrangement	2/22	8.2
Block design	2/22	8.5
Object assembly	5/21	7.9
Mazes	1/22	9.6
Symbols	3/22	7.9

SD = standard deviation.

IQ = Intellectual Quotient

**Figure 1:**
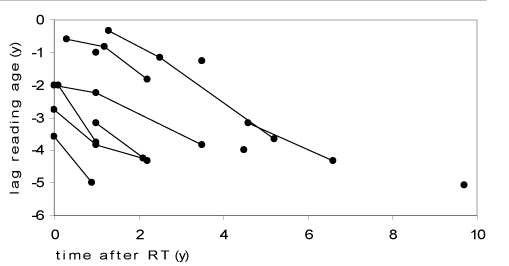
**Reading performances**. Differences between chronological age and reading age in years of 12 patients at different time points after therapy (4 had one test, 8 had sequential testing); Results of individual patients are connected with lines.

All of the 12 tested patients showed impairment in their reading skills, with a lag of 1 to 5 years between "reading age" and chronological age (median 3.8 years). The discrepancy was growing with time in all 8 patients with sequential testing (figure 1). Concerning visuospatial capacities, 3 of 16 patients had severe difficulties in reproducing the Rey-Osterrieth complex figure (mean Z-score of the whole group being -1.01 SD) while none of 11 patients tested had severe difficulties with the benton line orientation test (mean Z-score of the whole group being 0.56 SD). Short term memory measured by digit span was significantly diminished in 1 of 10 tested patients (mean Z-score of the whole group being-0.62 SD) and long term memory measured by word list was significantly diminished in 3 of 19 tested patients (mean Z-score of the whole group being -0.92 SD). Overall results of 12 patients who received the wisconsin card sorting test (WSCT) were within average limits, but 7 of these 12 showed attentional deficits with slow adaption, the tendency to keep one strategy, difficulties with reasoning, and problems to maintain the intentional thread. Difficulties within the WSCT were not correlated to the IQ scores.

Except reading skills, none of the tests showed declining results over time after therapy.

### Risk factor analysis (see table 3)

**Table 3:** Medical history of the patients and full scale IQ scores at last evaluation.

pt	age	location	hydroc.	shunt	surg.	complications	age at RT	last test	neurology	last IQ
1	13.7 y	FV, obex	yes	no	GTR	no	14 y	17.8 y	normal	84
2	4.3 y	FV, obex	yes	VCS	GTR	no	5.8 y	11.1 y	CS grade 2	83
3	1.5 y	FV, right angle	yes	VCS	GTR	no	2.6 y	4.5 y	normal	85
4	7.7 y	FV	yes	EVD	GTR	no	7.8 y	10.1 y	normal	108
5	13.8 y	FV, obex	yes	EVD	GTR	no	13.8 y	16.2 y	normal	86
6	8.8 y	FV, roof	yes	EVD	STR	no	9.3 y	12.7 y	normal	81
7	14.2 y	FV, angles	no	no	GTR	no	14.4 y	19.7 y	normal	82
8	4.1 y	right angle	no	no	GTR	infection	4.3 y	14.9 y	normal	112
9	10.9 y	FV	yes	VCS	GTR	no	11 y	13.2 y	normal	93
10	3.5 y	FV	yes	no	GTR	no	3.7 y	12.3 y	normal	83
11	8.2 y	right angle	no	no	GTR	no	8.3 y	12 y	paresis VI+VII	93
12	3.7 y	FV, obex	yes	VP	STR	no	3.8 y	10.7 y	CS grade 1	68
13	3.2 y	FV	yes	no	STR	no	3.3 y	17.5 y	normal	104
14	2.5 y	FV	yes	VP	GTR	no	2.7 y	18 y	CS grade 2	70
15	13.5 y	FV	yes	VCS	GTR	no	13.7 y	17.7 y	normal	90
16	0.3 y	FV	yes	no	GTR	no	1.3 y	16.9 y	CS grade 3	75
17	2.5 y	FV, brainstem	yes	no	GTR	no	6.2 y	10.7 y	CS grade 3	71
18	4.5 y	FV	no	no	STR	no	4.6 y	15.8 y	normal	115
19	9.8 y	FV	yes	VP	GTR	no	10 y	15.1 y	ptosis	97
20	2.9 y	FV, right angle	yes	EVD	GTR	subdural eff.	3 y	10.3 y	CS grade 1	65
21	6.8 y	FV to C4	no	no	GTR	no	6.9 y	7.9 y	normal	86
22	8.2 y	FV to C2	no	no	GTR	no	8.3 y	12.8 y	nystagmus	111
23	12.5 y	FV	no	no	GTR	no	12.6 y	14.8 y	normal	108

RT = radiotherapy; IQ = Intellectual Quotient; Hydroc. = hydrocephalus. Presence of hydrocephalus was noted in patients with clinical signs of raised intracranial pressure associated with enlarged lateral ventricles and/or bulging of the third ventricle.

Surg. = extent of surgery; C2–C4 = 2^nd ^and 4^th ^cervical vertebra; EVD = external ventricle drainage; VCS = ventriculocisternostomy; VP = ventriculo-peritoneal shunt; GTR = gross total resection; STR = subtotal resection.

CS = cerebellar signs; presence of cerebellar syndrome (Ataxia, Dysmetria, Nystagmus) was graduated as mild, moderate, or severe according to the impact on daily activities by an independent physician unaware of the neuropsychological performance using Riva's rating scale [22].

#### Age

**Figure 2:**
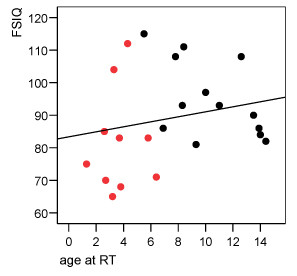
Of ten patients with age < 5 years at diagnosis, eight were irradiated before the age of 5. Regression line is also indicated (r = 0.22; p = 0.3). Black circles = patients > 5 y at diagnosis. Red circles = patients < 5 y at diagnosis.

Low IQ results occurred mainly in the young age group. Figure 2 shows the distribution of FSIQ values at last evaluation and age at irradiation. Comparing the IQ results of children younger than 5 years at irradiation with children who were older at the time of irradiation, the difference failed significance but there was a trend for poorer outcome in younger children. Mean FSIQ was 82.7 (n = 8, SD 17.2), and 92.5 (n = 15, SD 12.8) respectively (p = 0.1).

#### Interval since RT and FSIQ

Of 13 patients with a baseline evaluation, 10 patients were tested before the start of irradiation and three patients within the first year after completion of radiotherapy. One of these patients was below 3 years of age at diagnosis, therefore he received age adapted tests without IQ testing. Of the remaining 12 patients mean baseline FSIQ, VIQ and PIQ (SD) was 91.6 (10.6), 98.4 (8.9) and 85.8 (13.6). The only patient with a baseline FSIQ below 75 was diagnosed at 2.5 years and received delayed RT with neuropsychological evaluation and onset of irradiation at 5.9 years.

**Figure 3:**
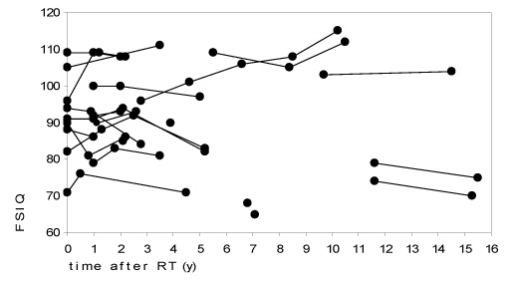
**FSIQ of the 23 patients at different time points after RT**. Results of individual patients are connected with lines.

In the longitudinal analysis there was no trend for loss of intellectual capacity over time after completion of irradiation. Figure 3 shows FSIQ scores of the sequential evaluations over time. There were six patients showing a decline in the measured IQ results, while five were gaining points. Of 11 patients who had baseline IQ testing and evaluation 2–5 years after radiotherapy (median 3.5 years) mean FSIQ (SD) at baseline and at last evaluation were 91.9 (11.0) and 91.3 (13.2), respectively. The difference between evaluations ranged between -10 to +12 points, with a median difference of 0.

#### Cerebellar syndrome and other influencing factors

**Figure 4:**
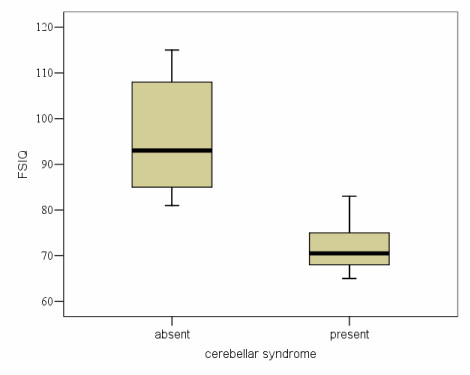
**FSIQ with absent (n = 16) and present (n = 6) cerebellar syndrome (CS) at time of neuropsychological evaluation**. Representation of the results is given as a Tukey and Cleveland's box-plot. The box represents the 5 principal centiles, ie 50% of the distribution. The line in the middle of the box represents the median. The line on top of the box joins the 90th centile. The line below the box joins the 10th percentile. Circles indicate the extreme values.

At the time of last neuropsychological evaluation 6 patients had a cerebellar syndrome. There was a strong correlation with decreased IQ scores. Mean FSIQ (SD) was 72.0 (6.3) within the group of children with persisting cerebellar syndrome compared to 95.2 (12.0) within the group of children showing no signs of cerebellar syndrome. This difference was highly significant (p < 0.001) (figure 4). Hand motor speed measured by Purdue pegboard evaluation was also highly correlated with FSIQ results (p = 0.003). With only 5 patients showing no signs of elevated intraventricular pressure (IVP) at initial presentation, the negative influence of elevated IVP was however significant (p = 0.025). Mean FSIQ with and without IVP at presentation was 83.9 (SD12.5) and 102.6 (SD14.4), respectively.

Patients with opposite lateral beams radiotherapy showed a lower mean FSIQ (SD) of 86.2 (16.7) compared to patients with conformal radiotherapy with FSIQ (SD) of 92.4 (12.6). The difference did not reach significance (p = 0.21).

#### Schooling

Three patients attended a regular school but had a delay of 2 or more years. One patient visited a special institute. The other patients were attending regular schools with no more than one year delay.

#### Endocrine deficits

Four patients had endocrinologic deficits which needed substitution (precocious puberty 2, growth hormone deficit 2). None of the patients had severe hearing impairment.

## Discussion

We conducted neuropsychological evaluations in patients with localised infratentorial ependymoma who received surgery and irradiation limited to the posterior fossa. Mean IQ scores evaluated with Wechsler IQ tests showed an overall moderate impairment but mean FSIQ remained in the normal range. While some patients had significant impairments in their PIQ scores, no significant impairment was seen in the VIQ score. Compared to previously published outcome scores of children who received whole brain irradiation these impairments were limited [16]. In our study IQ measures showed a high variability at all evaluated time points. Within the group which could be evaluated longitudinally (19/23), there was no trend for deterioration of intellectual functioning over time. This finding is in contrast to studies on medulloblastoma patients receiving CSI, who showed a deterioration of intellectual functions for years after the completion of therapy [7,23-25], and it is supporting the data by Merchant et al, who evaluated the influence of conformal RT for the treatment of ependymoma to intellectual outcome. With radiation limited to the tumour volume, they described stable intellectual functions with a median follow up of 3 years [3]. A recent report from Fouladi et al. also showed no significant longitudinal decline of IQ measures of patients with infratentorial tumours who received local RT compared to CSI [26].

With local posterior fossa irradiation, large parts of the supratentorial hemispheres and white matter are spared from irradiation, which might explain that there is no gradual IQ drop as it is seen after whole brain irradiation. Our data support this hypothesis since children receiving conformal RT tended to show a better outcome than those treated with opposite lateral beams. Merchant et al analysed with radiation dosimetry models that volume and dose of irradiation of the supratentorial brain was predictive for IQ in localised infratentorial ependymoma [27], which supports the above mentioned concept.

In our study very low IQ results were only observed in young children, but there was no statistical significant correlation between age at irradiation and intellectual outcome within our limited study population. While in different studies on patients who received CSI the progressive deterioration of neuropsychological functions was more pronounced in younger children [28,29], in our study there was no significant age dependent decrease of intellectual functions, and IQ results achieved at baseline evaluation and at follow up evaluations showed no difference. There was however a trend for worse outcome in younger children. But larger sample may be necessary to show a clear difference in outcome. Therefore we suppose that local posterior fossa RT is unlikely to be the only factor causing worse neuropsychological outcome in young children. As in our study, there were only 3 children, who were treated with radiotherapy before the age of 3, we are not able to draw definite conclusion about the role of very young age in the intellectual deficit after posterior fossa RT.

The intellectual deficits reported in our study might reflect also damages accrued by the disease and surgical therapy. This concept is supported by studies showing that IQ is impaired in survivors of posterior fossa tumours even in the absence of radiotherapy [22,30,31] suggesting contributing factors of the disease itself and surgical therapy on neuropsychological outcome.

Looking for other factors which could predict for low IQ performance we found a strong correlation of IQ and cerebellar damage, measured by the presence of cerebellar syndrome at the time of neuropsychological evaluation. A pivotal role of cerebellar damage for the presence of intellectual deficits was described recently by our group in a study evaluating 76 children with posterior fossa tumours, where disease factors and surgical complications were exceeding the negative effects of adjuvant therapy. Interestingly persistent cerebellar syndrome was more frequent in the latter described study population (51%) which consisted mainly of medulloblastoma patients, compared to this study (26%) [32].

Another factor which showed a trend to negatively influence the intellectual outcome in our study was hydrocephalus at presentation. Merchant et al analysed ventricular enlargement by MRI at different time points in patients with infratentorial ependymoma. They stated a relevant influence of hydrocephalus on intellectual achievement, while they postulated that the negative influence of ventricular enlargement was reversible if ventricular size decreases over time [33]. Since there was no regular longitudinal measurement in our cohort, we could not evaluate the influence of change in ventricular size.

Concerning the neuropsychological profile, the subtest analysis of the Wechsler IQ test showed impairments concerning processing speed and visual motor skills. Individual patients had reduced scores in subtests reflecting visual perceptive and memory problems, whereas the overall performance on these tasks was just slightly decreased. The impaired reading capacities may reflect problems with speed and possibly also visual problems. The battery of additional tests showed an increase of the lag between reading age and chronological age over time since therapy in all tested patients, which is likely due to a reduced rate of skill acquisition. This highlights that tests exploring reading skills are usefull read-outs for the monitoring of the outcome of these children. Furthermore there were individual deficits in visuospatial capacities, in attention and in memory functions. Similar deficits are described in patients suffering cerebellar astrocytoma [34-36] and medulloblastomas [16]. Although there seems to be a common spectrum of deficits, we like to emphasis, that there was a wide variability and that we couldn't detect a clear pattern of impairment. Possibly the diversity of impairments reflects the differing influence of perioperative and intraoperative damage done to the brain.

## Conclusion

In conclusion, our data show that intellectual functions are moderately impaired in survivors of infratentorial ependymoma. Compared to children who received CSI, neuropsychological outcome was favourable in children who received only local posterior fossa radiotherapy.

There was a wide variability of the level of intellectual achievements and specific impairments. The high variability is likely to be caused by cerebellar and cerebral damage reflecting the influence of disease and surgery-related factors. Studies looking at therapy optimization should include neurological and cognitive evaluations to further describe the influencing factors and possible mechanisms of intellectual impairment. This report also indicates that further refinement of adjuvant therapy for ependymoma should include means to deliver radiation with limited fields and better chemotherapies to defer radiotherapy in the youngest patients. Children should also be always monitored for neurological and neuropsychological outcome to ensure that they get the necessary support for rehabilitation.

## Competing interests

The author(s) declare that they have no competing interests.

## Authors' contributions

KVH participated in the design of the study, collected the data, performed the statistical analysis and drafted the manuscript.

VK conceived the study, participated in its design and coordination, performed and evaluated the neurocognitive tests, and helped in drafting the manuscript.

JLH participated performed the irradiation and evaluated the radiotherapy files.

CK conceived the study and followed the patients clinically.

GD conceived the study, participated in its design and coordination.

JG conceived the study, participated in its design and coordiantion, helped to collect the data, to perform the statistical analysis, and to draft the manuscript.

All authors read and approved the final manuscript.

## Pre-publication history

The pre-publication history for this paper can be accessed here:


